# Psychophysiological and Dual-Task Effects of Biofeedback and Neurofeedback Interventions in Airforce Pilots: A Pilot Study

**DOI:** 10.3390/s25082580

**Published:** 2025-04-19

**Authors:** Juan Pedro Fuentes-García, Juan Luis Leon-Llamas, Santos Villafaina

**Affiliations:** 1 Grupo de Investigación Análisis Didáctico y Comportamental del Deporte (ADICODE), Departamento de Didáctica de la Expresión Musical, Plástica y Corporal, Facultad de Ciencias del Deporte, Universidad de Extremadura, Avenida de la Universidad s/n, 10003 Cáceres, Spain; 2 Instituto Universitario de Investigación e Innovación en el Deporte, Universidad de Extremadura, Avenida de la Universidad s/n, 10003 Cáceres, Spain; svillafaina@unex.es; 3 Grupo de Investigación en Actividad Física, Calidad de Vida y Salud (AFYCAV), Departamento de Didáctica de la Expresión Musical, Plástica y Corporal, Facultad de Ciencias del Deporte, Universidad de Extremadura, Avenida de la Universidad s/n, 10003 Cáceres, Spain

**Keywords:** training, pilots, anxiety, autonomic modulation, performance, security, dual task

## Abstract

(1) Background: Neurofeedback (NFB) and biofeedback (BFB) have been shown to reduce stress, enhance physiological self-regulation, improve cognitive performance, and accelerate response times. Stimulating the sensorimotor rhythm (12–15 Hz) is particularly effective in improving working memory and selective attention. However, most studies on air force pilots focus on addressing post-traumatic stress disorder rather than investigating how these interventions might enhance performance and safety during flights, as explored in the present study. (2) Methods: Twelve Spanish Air Force fighter pilot trainees (mean age = 22.83 (0.94) years) participated in the study. Six pilots underwent 24 sessions of combined NFB and BFB training (experimental group), while six served as controls. (3) Results: The experimental group demonstrated improved heart rate variability during baseline, alarm sounds, math tasks, and real flights, which is indicative of greater parasympathetic modulation. A significant decrease in the Theta/SMR ratio was observed in the experimental group during the same conditions, suggesting improved focus, with lower values than the control group. Cognitive performance improved in the experimental group, with higher accuracy and a greater number of completed operations during math tasks. Regarding dual-task performance, the experimental group showed lower reaction time and a better ratio taps/reaction post-intervention. Psychological benefits included reduced cognitive, somatic, and state anxiety levels, along with increased self-confidence. (4) Conclusions: Neurofeedback and biofeedback training, integrated with real flights, simulators, and virtual reality, can enhance physiological regulation, cognitive performance, and emotional resilience, contributing to improved performance and safety in air force pilots.

## 1. Introduction

Cognitive load has been a fundamental aspect in the examination of aviation tasks since pilots are required to assimilate a vast amount of information from various sources [1,2]. These evaluations have contributed to the comprehension of the intricate and evolving mental demands of this operation [3,4]. Thus, given the multifaceted character of flying, a variety of psychophysiological techniques have been employed in this domain [5,6,7].

Different studies carried out with air force pilots have shown the high psychophysiological demands of certain flight situations. In this regard, previous studies have described that offensive and defensive aerial combat techniques can increase the perceived stress and effort awareness while lowering blood oxygen levels [8] and RR interval [9,10].

Despite the rigorous selection criteria for air force pilots, they are exposed to multiple stressors, including physical and mental strain, circadian rhythm disruptions, sleep deprivation caused by jet lag, and challenging environmental conditions [11]. These stressors contribute to an imbalance in immune-metabolic functioning, particularly among less experienced pilots, although this imbalance tends to improve with increased flight hours [11]. Similarly, studies on commercial airline pilots have shown that stress experienced before and during flight simulations can adversely affect performance, independent of carbon dioxide (CO2) levels [12]. Notably, greater heart rate variability (HRV) and a more balanced autonomic nervous system (ANS) function have been linked to a higher likelihood of successfully executing maneuvers [12].

Thus, the results of a study carried out with helicopter air force pilots in their first stages of training air force pilots concluded the effectiveness of the use of psychophysiological methods such as EEG and heart rate (HR) monitoring both in simulator as well as under real flight conditions for the development of an application that facilitated the instructors to decide which of the specific tasks to focus on during follow-up training [13]. As a consequence of the concern of the countries’ armies to provide their pilots with maximum safety and operational performance in flight, NASA developed an Aviation Safety Program in which a pilot BFB training in the cognitive awareness training study was included, showing that results differ between the control and experimental groups, with a greater reduction in the experimental group [14].

Human error accounts for 80–85% of aviation accidents, highlighting the critical need for interventions that enhance pilots’ psychophysiological resilience and operational performance under high-stress conditions [15]. Military and commercial pilots face unique cognitive and emotional challenges, requiring advanced strategies to manage stress and maintain optimal performance. Biofeedback (BFB) and neurofeedback (NFB) have emerged as promising tools to address these demands, with studies demonstrating their ability to improve stress resilience, cognitive functions, and physiological control. For example, HRV biofeedback has been shown to enhance pilots’ operational performance in as little as five minutes [15].

The regulation of Sensorimotor Rhythm (SMR; 12–15 Hz) is particularly relevant to pilot performance, as it enhances cognitive functions such as selective attention and working memory. SMR training has been linked to improved concentration [16], reduced anxiety, and better coping with competitive pressure [17]. Typically applied at electrode sites such as Cz or C4, SMR protocols aim to enhance SMR while suppressing Theta and fast Beta waves, promoting relaxation and attention [18]. Similarly, HRV biofeedback protocols, which incorporate rhythmic breathing techniques, have shown effectiveness in reducing stress and enhancing physiological control in high-demand populations, including elite athletes [19,20].

The Central Nervous System (CNS) plays a key role in regulating executive functions, attention, emotional control, and stress responses, with the anterior executive network acting as a hub for psychophysiological regulation [21,22]. HRV is widely recognized as a reliable biomarker for autonomic nervous system (ANS) functioning, with higher HRV values reflecting optimal balance and lower values indicating increased stress and cognitive load [23,24,25,26]. Among air force pilots, reduced HRV during real flight scenarios has been observed, suggesting the significant cognitive and emotional demands of their tasks [9,27].

Dual-task scenarios, such as combining flight simulations with Stroop tasks, further revealed the challenges faced by air force pilots. Studies indicated that novice pilots experience greater flight instability, exaggerated control forces, and lower reaction times under high G-force conditions (+3 Gz) compared to experts, underscoring the importance of psychophysiological training to handle these demands [28].

NFB has been employed since the 1960s by NASA for training astronauts and air force pilots, enabling individuals to self-regulate their EEG activity through real-time auditory or visual feedback [29]. Similarly, BFB, particularly HRV training, has demonstrated rapid effects on autonomic regulation, enhancing stress resilience and cognitive performance [30]. Despite the promising findings on the individual benefits of BFB and NFB, research on their combined application in air force pilots remains scarce. Most studies have focused on the treatment of traumatic brain injury [31] or post-traumatic stress disorder [32], leaving a significant gap in understanding their potential for optimizing flight performance under operational conditions.

HRV biofeedback has been shown to improve cognitive function and autonomic control within minutes [33], while SMR neurofeedback targets sensorimotor rhythm modulation, requiring more extensive training to induce lasting neurophysiological adaptations [34]. Evidence suggests that combining these interventions may yield complementary benefits: HRV biofeedback facilitates immediate autonomic stabilization, while SMR training enhances sustained cognitive control and attentional regulation [35]. Moreover, a systematic review on neurofeedback training protocols in sports highlighted that the efficacy of neurofeedback varies depending on the sport, the specific cognitive or motor skills targeted, and the level of athlete expertise [29]. SMR training has proven particularly effective in improving accuracy in disciplines requiring fine motor control and sustained attention, such as golf and rifle shooting. While novice athletes benefit from fundamental improvements in motor control and reaction times, elite athletes experience more subtle yet crucial enhancements, such as heightened focus and reduced anxiety [29].

Considering that the pilots in our study were still in training and therefore at a novice level, SMR training could be especially beneficial for precision tasks essential for flying an aircraft. Additionally, simultaneous SMR and HRV training could be particularly effective, as demonstrated by Shaw [30], who examined a ten-session SMR and HRV biofeedback program designed to optimize performance in Division 1 artistic gymnasts. The study found that gymnasts exhibited significantly improved balance beam performance during competition when biofeedback training was implemented compared to when it was discontinued. These findings suggest that HRV and SMR training may interact synergistically to establish an optimal pre-performance mind–body state, which is critical for high-stakes environments such as aviation [30].

Building upon these gaps, the present pilot study aims to evaluate the feasibility and potential impact of combined HRV biofeedback and SMR-based neurofeedback interventions on key psychophysiological markers and cognitive performance in air force pilots. Specifically, we hypothesize that these interventions will decrease sympathetic activation during real flights, enhance mathematical and dual-task performance, and improve SMR, HRV, and body temperature while reducing skin conductance, breathing frequency, and anxiety. Results would be relevant since aircraft pilots are a population particularly vulnerable to developing mental fatigue that harms, among other issues, mental focus [31].

## 2. Materials and Methods

### 2.1. Participants

A total of 12 pilots, 1 female and 11 males (age = 22.83 (0.94)) in the final stage of training from the Spanish Air Force participated in this experimental study (see Table 1). These 12 Second Lieutenants correspond to the total number that are selected each year to carry out the last phase of training to become pilots. All participants had two years of flying experience. These participants were randomized into two groups: experimental group (n = 6) and control group (n = 6) using a random number generator. According to the Declaration of Helsinki, all the procedures were approved by the University research ethics committee (approval number: 50/2024), and all participants agreed and gave written consent to participate in the study.

### 2.2. Procedure

#### 2.2.1. Initial and Final Evaluation

Full physiological profile: Before and after the intervention, the BioGraph Infiniti Multimodality Platform version 6.0., along with the 360 Suite software version 6.2.1. (Thought Technology, Montreal, QC, Canada), was used to conduct a full physiological profile. This assessment comprises eight distinct states: baseline (2 min), exposure to loud noise, in our case alarms sounds that can occur during the flight (2 min), rest (2 min), a mathematics task (2 min), rest (2 min), stressful memory (2 min), rest (2 min), and relaxation (3 min). The physiological profile was employed to evaluate pilots on their competence in managing stress [32]. In relation to this test, we were especially interested in knowing how the pilots behaved at the level of stress management before and after the training sessions in two tasks of the physiological profile that seem especially relevant to a fighter pilot. Firstly, “loud noise”, specifically, were reproduced during the test of all the alarms sounds that can occur in flight with the F5 aircraft used (bingo fuel, fire, landing gear, radar alert…), previously recorded from an operational F-5 M (Indra Company, Madrid, Spain) flight simulator. Secondly, the “math task” was used due to its direct relationship with calculation operations, which are important in fighter. In this way, experienced pilots appreciate the critical role that precise calculations, conversions, and computations play in ensuring safe and effective flight operations, even in the era of 21st-century technology. They routinely rely on these essential mathematical skills to interpret hold entries, evaluate aircraft range and performance data, assess crosswind components, plan descents, and apply a myriad of other crucial formulas that underpin successful flight management [33]. When the pilot arrives, after subtracting, for example, the number 5, he must take into account the concept of 360°, the calculation of degrees being fundamental during his flights, so the next correct number would be 330, which is calculated by subtracting the number 35 from the number 360 (325) and adding the number 5 to it, as it is the last number calculated by the pilot in his last subtraction operation. The following variables were assessed in the whole physiological profile tasks: breathing, conductance, temperature, HRV, and SMR, as well as the performance in the math task, considering the number of operations carried out by the pilot and the number of errors made.

Dual-task evaluation: A dual-task was designed to mimic the multiple tasks that pilots face during the flight (i.e., combine arm actions simultaneously with oral communication with the air base and with other colleagues who are flying). The dual task consisted of having to tap, from a total of 6 devices that lit up randomly, the light that lit up green as quickly as possible. Pilots were asked to achieve the greatest number of taps during 30 s, avoiding tapping the red lights. Simultaneously, participants had to say as many words as possible that were not proper names and verb conjugations. The words had to begin with the letter A or C depending on the letter assigned to each pilot based on a previous randomization. For this dual task, the BlazePod Trainer Kit system (BlazePod, Play Coyotta Ltd., Tel Aviv, Israel) was used.

Perceived anxiety, self-confidence, and HRV: HRV was analyzed during flights, as well as perceived anxiety and self-confidence levels before and after flights. The squadron leader chose two flight missions, one for the pre-flight and one for the post-flight, with similar levels of demands according to the training stage they were in. Participants were instructed not to consume any alcohol, coffee, or caffeinated drinks 24 h prior to the protocol in order to minimize any potential confounding effects. Not a single individual smoked or took cardioactive drugs like antidepressants, antipsychotics, or antihypertensives [34]. All flights were conducted between 8.00 and 14.00 h.

Resonance frequency: A resonance frequency test was performed using the BioGraph Infiniti Multimodality Platform and HRV Suite Software SA7580 (Thought Technology, Montreal, QC, Canada) prior to initiation of HRV in the eleventh session. For this test, the duration is 14 min, under which both the finger pulse and respiration signals are displayed on the screen below the respiration pacer, which automatically changes its pacing rate from 8 breaths/min to 5 breaths/min. It also lowers the pacer by 0.5 breaths/min every 2 min, and the pilot was encouraged to try and stick with the pacer as best she could throughout.

#### 2.2.2. BFB and NFB Intervention

##### Intervention Protocol and Materials

The duration of the training protocol with BFB and NFB was 16 weeks (starting on 23 November, 2023 and finishing on 14 March, 2024), with 1 session with BioGraph Infiniti every two weeks and 1 session with Evu TPS every week. In this way, the pilots alternately performed 1 session one week and 2 sessions the following week. NFB was applied using the BioGraph Infiniti EEG Suite version 6.1 software (Thought Technology, Montreal, QC, Canada). The EEG signal obtained from electrode position Cz was used for training. The NFB condition was treated with an SMR enhancement method that suppressed high beta (20–32 Hz) and theta (3.5–7.5 Hz) while enhancing SMR (12–15 Hz) [20,35]. The EEG signal obtained from electrode position Cz was used to train the protocols.

Likewise, the Evu TPS finger sensor was used with its own eVu-Senz application for Android (Thought Technology, Montreal, QC, Canada), which allows simultaneous monitoring of breathing, HRV, conductance, and temperature. By concurrently raising temperature and HRV and decreasing skin conductance, Evu Senz qualifies the capacity to induce a relaxing response, which has two training modes, rewards view mode and signal view mode. In this regard, more points are received during the session if more successful in generating this appropriate response is achieved. Additionally, the system may show the biosignals as success index values ranging from 0 to 100 and provides visual icon cues and feedback music to indicate when the biosignals are improving [32].

The pilots performed 24 work sessions with training in breathing, SMR, conductance, temperature, and HRV, 9 sessions using the BioGraph Infiniti, and 15 sessions using the Evu TPS. The 15 training sessions with Evu TPS comprised the following: (1) breathing (sessions 1, 3, 4, 6, and 7), (2) breathing + conductance (session 9 and 10), (3) breathing + HRV + conductance (sessions 12, 13, 15, and 16), (4) respiration + HRV + conductance + temperature + SMR (sessions 18, 19, 21, 22, and 24). The 9 training sessions with BioGraph Infiniti comprised the following: (1) breathing + SMR (sessions 2 and 5), (2) breathing + conductance + SMR (session 8), (3) breathing + HRV + conductance + SMR (sessions 11 and 14), (4) respiration + HRV + conductance + temperature + SMR (sessions 17, 20, and 23).

##### Training Sessions

Each of the work sessions was divided into two phases: (a) training in BFB and NFB and (b) transfer to real flight, with each of the two phases lasting a maximum of 50 min.

Phase A (BFB and NFB training):

The exercises in phase (a) were classified into the following categorizations based on training type for NFB or BFB:

1. SMR training in Cz using the BioGraph Infiniti Multimodality Platform and the 360 Suite (Thought Technology, Montreal, QC, Canada) (boat race). Three boats and three bar graphs are shown in the pilot’s visual field. Each boat moves forward when the signal from the matching bar graph is in the ON (or success state). The goal is to not let the other two boats (theta and high beta) get ahead and the center boat (SMR)—which is tied to the reward channel—get ahead. The presence of a green light (prize) or a red light (inhibit) after a boat crosses the finish line (right edge) indicates. the winner.
2. HRV preparing with BioGraph Infiniti Multimodality Stage and HRV Suite program (Thought Innovation, Montreal, QC, Canada) (archer shoots arrows at the target): Three prerequisites must be met for this screen to supply criticism: LF must be expanding (or steady), whereas VLF and HF must be dropping (or steady). The screen tracks the rate of add up to control values for VLF, LF, and HF. At this time, the animation starts to move forward, the soundtrack heightens, and focuses are accumulated. Keeping up the victory condition until the arrow hits the target is the pilot’s objective. When the condition is misplaced, the archer puts his arrow back into his quiver.
3. Training for respiration using the 360 Suite and the BioGraph Infiniti Multimodality Platform (Thought Technology, Montreal, QC, Canada) 360 Suite (slow breathing): Using a screen that shows a female balancing a ball behind her back at neck height, the goal is to educate the pilot to breathe steadily and gently at a rate of four to eight breaths per minute. If the breathing rate is recognized, the animation centers the ball, the music becomes louder, and points are accumulated. The pilot may close their eyes and enjoy the music and tones, as a tone corresponding to the signal value is audible when the breathing rate increases or decreases.
4. Using 360 Suite for arousal and temperature training while driving: Two graphs—one red for arousal and one blue for temperature—as well as a movie that simulates a pilot operating a vehicle are displayed on the computer screen. Each graph has a distinct but complementing piece of music. If the temperature increases and the arousal decreases, the automobile will begin to move, and the two music tracks would sound complete and complementary to one another.

The flight exercises in real flight, in a simulator, and in virtual reality in phase (b), transfer to flight through specific training, were planned by the squadron leader, being the same as those carried out by the control group. All missions consist of the following phases: (1) takeoff; (2) G-warm-up and G-awareness below FL 180; (3) air-to-air mission with two setups; (4) air-to-ground attack on the selected target; and (5) landing without visibility reduction. Both real and flight simulation protocols lasted 45 min.

A typical session of the intervention is summarized in Table 1.

**Figure 1:**
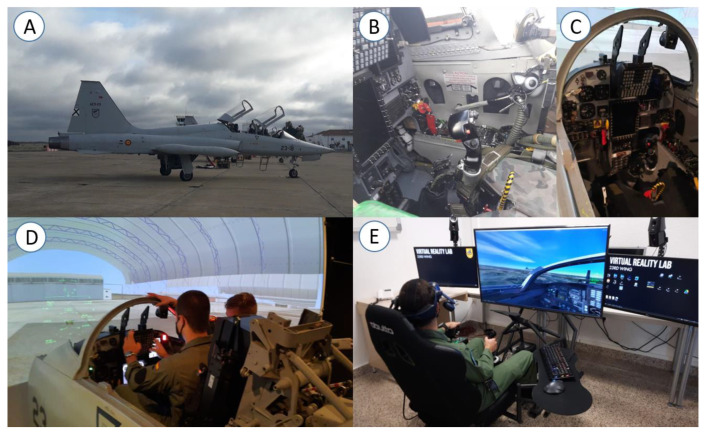
(**A**) F5 aircraft, (**B**) cabin of an F5 aircraft, (**C**) cabin of an F-5 M flight simulator, (**D**) F-5 M flight simulator, (**E**) virtual reality system Simulator F5 Flight Lab-Air Force and Space.

Phase B (Transfer to real flight):

In order to transfer the training to real flight situations but also to perform it with adequate levels of rest, the sessions were performed between 90 and 120 min before the briefing prior to each flight. Eight sessions were completed before real flight with an F5 aircraft, eight sessions before using the operational F-5 M (Indra Company, Madrid, Spain) flight simulator, and eight before using the virtual reality system Simulator F5 Flight Lab-Air Force and Space (Skydronexy, Badajoz Spain). This system comprises the Varjo XR-3 mixed reality headset, the HTC Controller 2.0 virtual reality headset, the Thrustmaster pendular rudder flight simulation expert rudder system + Thrustmaster hotas warthog throttle + joystick (Thrustmaster, Carentoir, France), the HTC Base Station 2.0 + the HTC Vive controller 2.0 (HTC Vive, New Taipei City, Taiwan). For this system, the HP OMEN 30L desktop computer with the following characteristics was used: Intel Core i9-11900K 64GB SSD 1TB PCIe × 2 NVIDIA RTX 3090 24 GB (Figure 1).

### 2.3. Instruments, Processing, and Outcomes

A quiet environment with regulated humidity and temperature (22.3 (1.0) °C; 46.4 (2.8%)) was used for the measurements. Pilots were advised not to consume any substances that impact the neurological system for 24 h before the intervention, which was carried out in the morning (between 10 a.m. and 12 p.m.).

#### 2.3.1. BFB and NFB Equipment

On the one hand, the BFB and NFB intervention was carried out using a ProComp Infiniti device (Thought Technology, Montreal, QC, Canada). Research on BFB training in athletes has previously made use of this 8-channel multimodality encoder for real-time computerized measurements [20,36,37]. Training, data analysis, and reports were conducted using the BioGraph Infiniti Multimodality Platform, BioGraph Infiniti EEG Suite and the 360 Suite and HRV Suite software (Thought Technology, Montreal, QC, Canada) (256 Hz). These programs integrate the traditional physiological BFB (arousal and peripheral temperature) with heart rate monitoring (HRV) and NFB (EEG BFB) protocols in a single package. The use of all these platforms was performed through a desktop computer with the following characteristics: Processor: AMD Ryzen Threadripper 2990WX (32 Core, 4.2 GHz, 64 MB Cache, 250 W); motherboard: gigabyte x399 aorus xtreme; graphics card: EVGA GeForce GTX 1060 6 GB; power supply: Corsair RM1000x; cooling: Corsair h100i v2 liquid cooling. A 27” LG 27MP60G-B was used as the second monitor. Likewise, the Evu TPS finger sensor was used with its own eVu-Senz application for Android, which has two training modes, rewards view mode and signal view mode (Thought Technology, Montreal, QC, Canada), using a Samsung Galaxy Tab S6 Lite—10.4” tablet (Qualcomm Snapdragon 720G Processor, 4 GB RAM, 128 GB Storage, Wi-Fi, Android 12), which allows simultaneous monitoring of breathing by having a gyroscope that infers breathing through movement, HRV, conductance, and temperature biosignals.

#### 2.3.2. Heart Rate Variability (HRV) During Flight Tests

A heart rate monitor (Polar RS800CX, Oy, Kempele, Finland) was used to record the HRV [38], and Kubios HRV software (v. 3.3) was used for analysis [39]. This study adhered to the Task Force’s recommendations from the North American Society of Pacing and Electrophysiology [40] and the European Society of Cardiology. The heart rate monitor’s collected RR data were transferred to the Kubios HRV program.

#### 2.3.3. Dual Tasks Equipment

The BlazePod Trainer Kit system (BlazePod, Play Coyotta Ltd., Tel Aviv, Israel) was used to perform the dual-task. The BlazePod app was used in a Xiaomi Redmi Note 8 mobile phone, Qualcomm Snapdragon 655 octacore processor, 2.0 GHz, RAM 4 GB, ROM 64 GB, Android 9.0 (Xiaomi Corp. Beijing, China) to record the number of taps made by each pilot during the 30 s as well as the reaction time. Moreover, all tests were recorded on video with a Sony Handycam 4K FDR-AX43 camera (Sony Group Corporation, Tokyo, Japan) to verify the number of presses as well as the number of correct words pronounced during the test.

#### 2.3.4. Cognitive Anxiety, Somatic Anxiety, Self-Confidence, and State Anxiety Before the Flight

The pilots’ pre-competitive anxiety was measured using the Competitive State Anxiety Inventory–2R (CSAI-2R) [41], Spanish version [42]. Self-confidence, somatic anxiety, and cognitive anxiety may all be extracted from the 17 items in this questionnaire. A Likert answer structure is used to evaluate each issue, offering four alternatives on a 4-point scale ranging from “not at all” to “very much so”. The purpose of the Cognitive Anxiety sub-scale is to measure negative emotions regarding performance and its aftermath. It has five things and a total score between five and twenty points. Seven items make up the Somatic Anxiety subscale, which measures how people perceive bodily signs of anxiety such as tense muscles, elevated heart rate, and sweating.

The State Anxiety Inventory (STAI-S), Spanish version [43], which has 20 questions on a single scale and enables the investigation of anxiety phenomena, was also used to quantify anxiety. The questionnaire’s 20 items are scored on a Likert scale that ranges from 0 (almost never) to 3 (nearly often). A comparatively steady anxious propensity, where the propensity to see events as hazardous and subsequently generate anxiety, is shown by the scale, which depicts the participant’s feelings at a “specific instant”. Then, 30 is added to the result after subtracting the negative scale from the positive scale.

### 2.4. Data Processing

The NFB and EEG equipment used included a ProComp Infiniti differential amplifier and software (Thought Technology Ltd., Montreal, QC, Canada). Electrodes were placed on individualized target areas for the Ind-NFB group, while for the St-NFB group, they were positioned at the Pz location according to the international 10–20 system. The reference electrode was attached to the left earlobe, and the ground electrode was placed on the right earlobe. Electrode sites were prepped using an abrasive conductive gel (Neurprep, Weaver and Company, Aurora, Colorado, U.S.), and a conductive paste (ac cream, Spes Medica s.r.l., Genova, Italy) was applied to the electrode cups to ensure that impedance remained below 5 kΩ. The raw EEG signal was recorded at a sampling rate of 256 Hz and converted online via an A/D process for real-time feedback. BioGraph Infiniti software version 6.0. applied infinite impulse response (IIR) filters to the recorded signal to extract frequency-domain features. Spectral amplitude estimates were computed for the active electrode site using raw 1 s EEG segments, achieving a frequency resolution of 1 Hz up to 30 Hz. Bandpass filters were used to extract theta/SMR ratio (theta: 3.5 to 7.5 Hz; SMR: 12 to 15 Hz) using fast Fourier transformations.

HRV measurements were obtained using a BioGraph Procomp Infinity 6.1™ (Thought Technology) device with a sampling rate of 2048 Hz. This system includes an internal, user-activated calibration feature that ensures high-quality signal acquisition while avoiding the costly downtime associated with factory recalibration. Participants’ standard electrocardiography (ECG) recordings were collected using an ECG-Flex/Pro sensor. The built-in filters and CardioPro Infinity software SA7590 were used to normalize ECG signals, power line interference at 50 Hz, electrode-related noise, movement artifacts, muscle contractions, respiration, and electrical disturbances from other devices.

CardioPro Infinity software was employed to acquire HRV signals, identify R-peaks within the QRS complexes of ECG recordings, and determine RR intervals. ECG artifact detection followed a three-step procedure: First, a 5 s window was applied to visually inspect raw ECG signals and interbeat intervals (IBI). Second, IBI data normalization was performed using three complementary techniques to identify ECG artifacts: (a) merging short IBI values to account for extra beats, (b) splitting IBI values into two equal segments for missed beats, and (c) averaging consecutive IBI pairs that included both long and short intervals. This approach was selected to maintain the continuity of the IBI series, which was essential for frequency-domain analysis. Finally, the HRV analysis module of CardioPro Infiniti was used to assess HRV indices in both time and frequency domains.

To process real flight HRV measurements, an intermediate filter was used to address potential artifacts. When compared to the average of the preceding beats, RR intervals that were shorter or longer than 0.25 s were automatically detected and substituted with cubic spline interpolation [44,45]. Non-linear, frequency-domain, and time-domain measurements were extracted using Kubios software (v. 3.3) [39]. Mean heart rate (HR), RR intervals, RR50 count divided by the total number of all RR ranges (pNN50), the standard deviation of all RR intervals (SDNN), and the square root of differences between consecutive RR intervals (RMSSD) were all included in the time domain. Very low-frequency (VLF, 0–0.04), low-frequency (LF, 0.04–0.15 Hz), and high-frequency (HF, 0.15–0.4 Hz), as well as the ratio (LF/HF), were computed in the frequency domain. The sample entropy (SampEn), detrended fluctuation analysis (DFA), the RR variability from heartbeat to short-term Poincaré graph (width) (SD1), and the RR variability from heartbeat to long-term Poincaré graph (length) (SD2) were extracted with respect to non-linear measures.

### 2.5. Statistical Analysis

The SPSS statistical package (Statistical Package for Social Sciences, version 25 for Windows, IBM Corporation, Armonk, NY, USA) was used to do the statistical analysis. Non-parametric tests were used after the Shapiro–Wilk test results.

To investigate differences within and between groups, non-parametric statistics were used. To compare the pre- and post-measures for every variable in the experimental and control groups, the Wilcoxon signed-rank test was employed. Mann–Whitney U test was used to explore the differences between experimental and control group in all study variables. Spearman’s rho correlation coefficients were calculated to explore the relationships between changes in theta/SMR ratio and physiological variables (post-intervention vs. pre-intervention) within each group (NFB and BFB and Control). For the non-parametric tests, effect sizes [r] were computed and categorized as follows: 0.5 indicates a high effect, 0.3 indicates a medium effect, and 0.1 indicates a minor effect [46,47]. The Benjamini–Hochberg method was used to adjust the *p*-value in order to manage the false discovery rate [48].

## 3. Results

Table 2 shows the descriptive data of participants in age, years of flying experience, height, weight, and body mass index (BMI). Differences were not found in the baseline.

Table 3 shows the pre- and post- values of the full-physiological profile. Within-group analyses showed that the experimental group significantly reduced the skin conductance and respiratory rate after the intervention (*p*-value < 0.05). Moreover, SDNN, LF/HF and pNN50 significantly increased after the intervention (*p*-value < 0.05). In addition, the Theta/SMR ratio was significantly reduced after the intervention (*p*-value < 0.05). The control group did not experience significant differences between pre and post in any of the variables. Between-group analyses showed significant intervention effects, reducing the breaths per minute, increasing values of SDNN and LF/HF, as well as a lowering Theta/SMR ratio (*p*-value < 0.05).

Table 4 shows the pre- and post- results of the alarm sounds of the full-physiological profile. Within-group analyses showed that the intervention group significantly reduced the skin conductance and respiration rate after the intervention (*p*-value < 0.05). In the same line, the heart rate decreased while SDNN and LF/HF increased after the intervention (*p*-value < 0.05). Moreover, the Theta/SMR ratio was reduced after the intervention (*p*-value < 0.05). The control group did not experience significant differences between pre and post in any of the variables. Between-group analyses showed that the experimental group, compared to the control group, showed significant differences after the intervention with lower values in skin conductance and breaths per minute, as well as higher values of SDNN and LF/HF (*p*-value < 0.05).

Regarding correlational analyses, in the NFB and BFB group, the Theta/SMR ratio change showed a strong negative correlation with pNN50 change (r = −0.943, *p* < 0.01). Additionally, the Theta/SMR ratio change was positively correlated with the number of taps change (r = 0.812, *p* < 0.050). Correlations in the control group were not found.

Table 5 shows the pre- and post- results of the math task state of the full-physiological profile. Within-group results showed that the experimental group significantly reduced the skin conductance and respiration rate after the intervention (*p*-value < 0.05). Moreover, heart rate significantly decreased, while SDNN significantly increased after the intervention (*p*-value < 0.05). In addition, the Theta/SMR ratio was reduced after the intervention. Moreover, the number of correct operations increased while the % of incorrect operations after the intervention is reduced. The control group did not experience significant differences between pre and post in any of the variables. Between-group analyses showed that the experimental group, compared to the control group, showed significant differences after the intervention with lower values in skin conductance and breaths per minute, higher values of SDNN, as well as a lower Theta/SMR ratio (*p*-value < 0.05). In addition, the experimental group showed a significantly higher number of correct operations and a lower % of incorrect operations after the intervention than the control group (*p*-value < 0.05).

Regarding correlational analyses, in the NFB and BFB group, the change in the Theta/SMR ratio was positively correlated with the change in the somatic anxiety (r = 0.841, *p* < 0.036). In the control group, the Theta/SMR ratio change showed a negative correlation with SDNN change (r = −0.900, *p* < 0.037).

**Figure 2:**
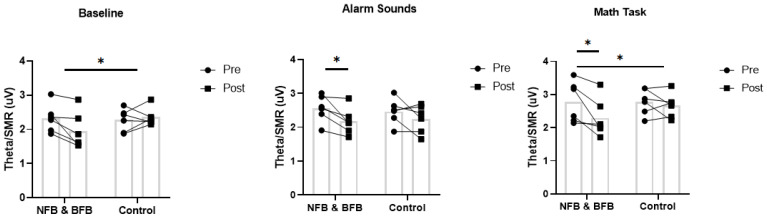
Intragroup and intergroup differences in the Theta/SMR variable in the three most representative tasks of the full psychological profile. * *p*-value < 0.05.

Figure 2 shows more details of the evolution of Theta/SMR in each of the participants of the control and experimental group for each of the full physiological profile conditions (baseline, alarm sounds, and math task).

Table 6 shows the results of the intervention in the dual-task performance of pilots pre- and post-intervention. Within-group results showed that the experimental group presented lower values of reaction time during the dual-task and as well as a higher ratio tap/reaction time after the intervention (*p*-vale < 0.05). The control group did not experience significant differences between pre and post in any of the variables. Between-group results showed that there were no significant differences between the experimental group and the control group in any of the variables.

Regarding correlational analyses, in the NFB and BFB group, the Theta/SMR ratio change was negatively correlated with Skin conductance change (r = −0.886, *p* < 0.019). Correlations in the control group were not found.

Table 7 shows the pre- and post- values of anxiety and self-confidence levels prior to the real flight. The experimental group showed significant lower values of cognitive anxiety, somatic anxiety, and state anxiety after the intervention, while self-confidence values increased. The control group did not experience significant differences between pre and post in any of the variables. Between-group analyses showed that the experimental group, compared to the control group, presented significant differences after the intervention with higher values of somatic anxiety (*p*-value < 0.05).

Table 8 shows the differences between pre and post flight in the HRV measurements. Within-group differences were not detected in any of the variables investigated. However, between-group differences were observed in SDDN (*p*-value = 0.043) and SD2 (*p*-value = 0.043). In this regard, SDNN and SD2 increased after the NFB and BFB intervention, whereas they were reduced in the control group.

## 4. Discussion

This study aimed to evaluate the effects of BFB and NFB interventions on air force pilots. Our hypotheses proposed that BFB and NFB would increase SMR during cognitive tasks, exposure to alarm noises, and real flights, while reducing sympathetic modulation (evidenced by higher HRV and lower skin conductance and respiratory frequency). Additionally, we hypothesized that pilots undergoing these interventions would experience reduced anxiety and increased self-confidence before real flight scenarios.

NFB operates within the framework of learning and performance enhancement by leveraging the brain’s inherent capacity for neuronal plasticity through operant conditioning [49]. During NFB training, individuals receive real-time feedback that enables them to modulate specific neural activities, thereby inducing neuromodulatory effects that modify relevant neural networks. This process not only strengthens neural pathways associated with desired cognitive and emotional states but also promotes long-term synaptic enhancements through mechanisms such as long-term potentiation (LTP)—the enduring increase in synaptic efficacy resulting from repeated high-frequency stimulation [50]. For example, training to increase SMR activity (12–15 Hz), which is associated with a relaxed yet focused state, has been linked to improvements in attention and working memory [51]. By fostering both immediate neuromodulatory changes and consolidated synaptic modifications via LTP, NFB contributes to better cognitive control and reduced anxiety, outcomes that are critical for optimal performance in high-demand environments such as aviation.

Following the intervention, the experimental group demonstrated a significant reduction in the theta/SMR ratio across baseline, alarm sound, and math task conditions, with no changes observed in the control group. Furthermore, post-intervention comparisons between groups revealed significantly lower theta/SMR ratios in the experimental group during baseline and math tasks. These findings align with prior research reporting similar reductions in theta/SMR ratios and psychological symptoms after NFB interventions, such as those conducted on fibromyalgia patients [52]. Consistent with our results, a study with elite chess players showed decreased theta/SMR ratios in response to loud noises following a similar BFB and NFB program [20]. Such reductions could suggest enhanced cognitive regulation and a better ability to inhibit theta activity while increasing SMR, crucial for maintaining attention during cognitive and emotional stressors [53]. In addition, the reduction in theta/SMR ratios during math tasks observed in this study supports the notion of improved cognitive processing efficiency following NFB training. Previous research on amateur boxers revealed that individuals with lower theta/SMR ratios exhibit superior concentration and attentional control compared to control groups, highlighting the potential of such interventions to enhance task-specific cognitive abilities [54]. Furthermore, the results from the NFB and BFB group show interesting patterns regarding the Theta/SMR ratio and its relationship with various physiological and performance measures. During baseline, the strong negative correlation between Theta/SMR and pNN50 (%) (r = −0.943, *p* < 0.01) suggests that increased SMR activity is linked to enhanced parasympathetic modulation, supporting the role of SMR training in improving autonomic control and stress resilience, as suggested by previous research on neurofeedback training [55]. The positive correlation with the number of taps (r = 0.812, *p* < 0.050) further implies that improvements in SMR may facilitate motor performance, which is consistent with findings showing that SMR training can enhance sensorimotor integration and reaction time [56,57].

Our findings emphasize the utility of SMR-based NFB in optimizing cognitive performance for the demanding mathematical and computational tasks required of air force pilots [33,58,59]. BFB interventions, particularly those targeting heart rate variability (HRV), enable individuals to gain voluntary control over autonomic functions. By providing feedback on physiological parameters such as heart rate, skin conductance, and respiration rate, BFB promotes autonomic balance, characterized by increased parasympathetic (vagal) activity and reduced sympathetic arousal. In this regard, we observed between-group differences in SDNN and SD2. This could suggest that the BFB NFB intervention may enhance autonomic flexibility, cardiovascular adaptability, stress resilience and emotional regulation in air force pilots. This is essential for pilots operating in high-stress environments. In the same line, studies have demonstrated that HRV biofeedback can lead to significant improvements in autonomic regulation, contributing to better performance under pressure [49]. SDNN reflects overall HRV, while SD2 represents long-term HRV components, both of which are associated with the autonomic nervous system’s capacity to respond to stressors [60]. An increase in these parameters post-intervention could indicate improved autonomic regulation, which is crucial for pilots operating in high-stress environments. These findings align with previous research suggesting that HRV can serve as a biomarker of mental health resilience [61], highlighting the potential of HRV BFB in enhancing physiological and psychological well-being. However, further research with larger cohorts is warranted to confirm these preliminary findings and to explore the underlying mechanisms contributing to the observed improvements in HRV parameters.

BFB training targeting respiration, arousal, and HRV also yielded improvements in autonomic regulation. Post-intervention, the experimental group showed higher SDNN values at baseline, alarm sound, and math task conditions, along with increased LF/HF ratios and reduced respiratory rates. These changes were absent in the control group, underscoring the efficacy of the intervention. Similar benefits of BFB on HRV and breathing coherence have been reported in athletes and under cognitive stressors [62,63]. Improvements in autonomic adaptability may enhance stress resilience, a critical requirement for high-pressure aviation environments [17]. Moreover, skin conductance results mirrored HRV improvements, with the experimental group exhibiting significantly lower conductance values at baseline, during alarm sounds, and math tasks. These findings are consistent with prior research showing BFB and NFB effectiveness in mitigating arousal during cognitive stress [63,64]. However, contrary to previous studies linking reduced body temperature to stress processing [17], no significant changes in temperature were observed in this study. Thus, this discrepancy warrants further investigation.

Cognitive performance, assessed through math tasks, improved significantly in the experimental group, with a higher number of correct operations and fewer errors post-intervention. These results align with prior studies demonstrating the benefits of BFB and NFB on attentional vigilance, spatial ability, and cognitive performance [65,66]. Moreover, dual-task performance improved in the experimental group, reflected by faster reaction times and higher taps/reaction ratio. Similar findings have been observed in air force pilots, where dual-task training enhanced working memory and attentional control [67]. In this regard, both NFB and BFB facilitate improved self-regulation by allowing individuals to become more aware of their physiological and neural states. This heightened awareness enables better control over cognitive processes and emotional responses. For example, NFB training aimed at increasing SMR has been associated with enhanced inhibitory control, allowing individuals to suppress irrelevant or distracting information more effectively. This mechanism is particularly beneficial in complex tasks requiring sustained attention and quick decision-making, such as piloting aircraft [51]. Given the critical role of dual-tasking in aviation, these findings highlight the potential of BFB and NFB to reduce human decision errors, a major contributor to aviation accidents [68].

Regarding anxiety and self-confidence, the experimental group reported reduced cognitive, somatic, and state anxiety levels alongside increased self-confidence post-intervention. These findings are consistent with systematic reviews emphasizing the role of BFB and NFB in managing anxiety and enhancing mental preparation in athletes [19,69]. In particular, HRV enhancement is known to decrease physicological markers of stress, leading to a calmer mental state [49]. This reduction in anxiety not only improves overall well-being but also enhances cognitive performance by preventing the detrimental effects of stress on attention and memory. This aligns with the observed correlations between the Theta/SMR ratio and somatic anxiety (r = 0.841, *p* < 0.036), indicating that increased SMR activity was linked to heightened somatic arousal, suggesting a more complex relationship between SMR training and physiological anxiety responses. While SMR training has been shown to improve autonomic control, its potential interaction with somatic anxiety warrants further investigation, particularly in high-pressure environments like flight operations. This complexity is also reflected in the correlation found between the Theta/SMR ratio and other physiological measures such as skin conductance and respiration rate, reinforcing the notion that autonomic regulation may interact with anxiety in non-linear ways.

While most previous studies in the field of NFB and BFB have employed well-established indices of cognitive and autonomic regulation—such as reaction time, EEG, HRV, or even math tasks—these measures do not fully capture the complex motor and cognitive demands unique to aviation. To address this gap, we evaluated autonomic modulation and EEG responses under conditions designed to mimic aviation-specific stressors: listening to alarm noises, performing a specialized math task, and engaging in a dual-task paradigm that combined a mental challenge with a motor task. In real flight operations, pilots must continuously acquire and update internal models to predict and adapt to rapidly changing conditions, especially when visual feedback is limited and feedforward control is critical [70]. It is plausible that the enhancements in SMR activity and autonomic balance observed in our study not only improve general cognitive performance but also facilitate the formation of these internal models. Improved internal model acquisition could lead to better anticipatory control and motor coordination, thereby reducing reaction times and decision errors in flight scenarios. Future studies might incorporate flight simulation or virtual reality environments that more directly replicate actual flight demands, providing a clearer link between the neurophysiological improvements observed here and operational performance in air force pilots.

This study has several limitations. First, the relatively small sample size of 12 air force pilots may limit the generalizability of the findings. Although this sample represents the entire cohort undergoing advanced training in a given year in Spain, the limited number of participants restricts the ability to draw broad conclusions. Second, the use of a passive control group that did not receive any intervention is a significant limitation. This choice makes it difficult to assess the specific effects of the combined biofeedback (BFB) and EEG-based neurofeedback (NFB) training, as it does not allow for differentiation between the effects of BFB and NFB individually. Despite these limitations, this pilot study provides a valuable foundation by demonstrating the applicability of BFB and NFB technologies among air force pilots and offering replicable procedures to assess their effectiveness in other domains. The preliminary findings suggest that these interventions could be effective tools for enhancing performance, underscoring the importance of conducting future studies with larger sample sizes and more rigorous experimental designs to validate these initial results.

## 5. Conclusions

In this pilot study, the combination of neurofeedback (NFB) and biofeedback (BFB) training with real flights, simulators, and virtual reality appeared to improve physiological and psychological responses in air force pilots. The experimental group exhibited reduced skin conductance and respiratory rates in response to warning sounds and math tasks alongside decreased anxiety and increased self-confidence before flights. Heart rate variability (HRV) analysis revealed greater parasympathetic modulation during baseline, alarm sounds, math tasks, and real flights, with enhanced values compared to the control group. Additionally, a reduced Theta/SMR ratio indicated enhanced focus in the experimental group. Cognitive performance improved, with more operations completed and fewer errors during math tasks, outperforming the control group. The experimental group also showed lower cognitive, somatic, and state anxiety and higher self-confidence, suggesting the intervention’s effectiveness in enhancing performance and stress regulation. However, given the preliminary nature of this study and its inherent limitations, these findings should be interpreted with caution. Further research with larger sample sizes and more rigorous experimental designs is necessary to validate these initial observations.

## Figures and Tables

**Table 1:** Example of an NFB and BFB session for the pilot.

Phase A Exercises (45 min)	Phase B Exercises (45 min)
Breathing: breathing slowly, already described (5 min). HRV: archer shoots arrows at the target, already described (10 min). Arousal and temperature: driving a car, already described (5 min). SMR: boat race, already described (30 min).	Individual takeoff. G- warm up/G- awareness below FL 180. Air–air mission with two set-ups. Air–ground attack on the selected target. Landing without a reduction in visibility.

Note: This session corresponds to session 14 of the intervention program.

**Table 2:** Descriptive characteristics of the participants.

Participants	Control Group Mean (SD)	Experimental Group Mean (SD)	*p*-Value
Age (years)	22.33 (0.52)	23.33 (1.03)	0.132
Flying experience	2	2	1
Height (cm)	180 (4.29)	179.33 (5.82)	0.937
Weight (kg)	80.33 (4.72)	76 (7.24)	0.310
BMI (kg/m2)	24.78 (0.64)	23.60 (1.44)	0.180

SD: Standard Deviation.

**Table 3:** Pre- and post-intervention effects of the full-physiological profile during baseline.

Variables				Within Group Comparison			Between Group Comparison		
		Pre Mean (SD)	Post Mean (SD)	Z	*p*-Value	Effect Size	Z	*p*-Value	Effect Size
Skin conductance (μS)	NFB and BFB	3.70 (2.37)	2.18 (1.11)	−1.992	0.046 *	0.813	−1.278	0.201	0.369
	Control	2.37 (1.48)	2 (0.86)	−1.214	0.225	0.496			
Respiration rate (breaths per minute)	NFB and BFB	12.32 (2.59)	6.05 (0.53)	−2.201	0.028 *	0.900	−2.008	0.045 *	0.580
	Control	12.59 (3.18)	11.26 (2.03)	−0.674	0.500	0.275			
Temperature (°C)	NFB and BFB	32.12 (2.07)	32.53 (2.55)	−1.572	0.116	0.642	−1.095	0.273	0.316
	Control	30.64 (3.27)	30.68 (3.84)	−0.135	0.893	0.055			
Heart rate (beats/min)	NFB and BFB	65.28 (6.97)	62.65 (5.48)	−1.153	0.249	0.062	−1.461	0.144	0.422
	Control	63.77 (9.42)	68.43 (9.04)	−0.944	0.345	0.385			
SDNN (ms)	NFB and BFB	93.68 (18.90)	150.17 (54.12)	−2.201	0.028 *	0.900	−2.008	0.045 *	0.236
	Control	102.67 (39.84)	99.11 (33.45)	−0.674	0.500	0.275			
LF/HF	NFB and BFB	4.41 (2.42)	18.10 (8.28)	−2.201	0.028 *	0.900	−2.739	0.006 *	0.791
	Control	2.33 (3.17)	1.42 (0.44)	−0.135	0.893	0.055			
pNN 50 (%)	NFB and BFB	24.12 (6.98)	31.88 (4.82)	−2.201	0.028 *	0.900	−0.365	0.715	0.105
	Control	21.22 (8.94)	23.22 (10.83)	−0.405	0.686	0.165			
Theta/SMR (uV)	NFB and BFB	2.32 (0.41)	1.95 (0.54)	−2.207	0.027 *	0.901	−2.379	0.017 *	0.687
	Control	2.27 (0.33)	2.36 (0.29)	−1.214	0.225	0.496			

SD: Standard Deviation; * *p*-value < 0.05.

**Table 4:** Pre- and post-intervention effects of the full-physiological profile while listening state alarm sounds.

Variables				Within-Group Comparison			Between-Group Comparison		
		Pre Mean (SD)	Post Mean (SD)	Z	*p*-Value	Effect Size	Z	*p*-Value	Effect Size
Skin conductance (μS)	NFB and BFB	4.62 (3.08)	2.82 (1.75)	−2.201	0.028 *	0.900	−2.008	0.045 *	0.580
	Control	2.85 (1.97)	2.92 (2.18)	−0.271	0.786	0.111			
Respiration rate (breaths per minute)	NFB and BFB	12.57 (3.27)	6.76 (132)	−2.201	0.028 *	0.900	−2.008	0.045 *	0.580
	Control	13.85 (2.03)	12.78 (2.48)	−1.214	0.225	0.496			
Temperature (°C)	NFB and BFB	32.56 (1.26)	33.04 (1.84)	−0.365	0.715	0.149	−0.245	0.806	0.071
	Control	30.58 (2.82)	30.58 (3.73)	−0.405	0.686	0.165			
Heart rate (beats/min)	NFB and BFB	73.69 (4.61)	69.54 (2.85)	−2.201	0.028 *	0.900	−1.643	0.100	0.474
	Control	69.45 (6.63)	72.58 (8.71)	−0.674	0.500	0.275			
SDNN (ms)	NFB and BFB	79.99 (35.48)	125.63 (40.98)	−2.201	0.028 *	0.900	−2.191	0.028 *	0.632
	Control	84.57 (27.98)	79.83 (16.74)	−1.214	0.225	0.496			
LF/HF	NFB and BFB	3.28 (1.33)	7.39 (3.18)	−2.201	0.028 *	0.900	−2.191	0.028 *	0.632
	Control	1.41 (089)	2.30 (2.06)	−0.944	0.345	0.385			
pNN 50 (%)	NFB and BFB	13.60 (10.68)	16.62 (9.12)	−0.943	0.249	0.385	−0.548	0.584	0.159
	Control	17.45 (8.07)	18.74 (7.03)	−0.674	0.500	0.275			
Theta/SMR (uV)	NFB and BFB	2.56 (0.39)	2.18 (0.39)	−2.201	0.028 *	0.900	−1.461	0.144	0.422
	Control	2.46 (0.38)	2.25 (0.46)	−0.552	0.581	0.225			

SD: Standard Deviation; * *p*-value < 0.05.

**Table 5:** Pre- and post-intervention effects of the math task of the full-physiological profile pre and post intervention.

Variables				Within-Group Comparison			Between-Group Comparison		
		Pre Mean (SD)	Post Mean (SD)	Z	*p*-Value	Effect Size	Z	*p*-Value	Effect Size
Skin conductance (μS)	NFB and BFB	5.69 (3.44)	3.09 (1.40)	−2.201	0.028 *	0.900	−2.191	0.028 *	0.632
	Control	3.98 (231)	4.28 (2.63)	−0.135	0.893	0.055			
Respiration rate (breaths per minute)	NFB and BFB	13.41 (1.67)	10.19 (1.75)	−2.201	0.028 *	0.900	−2.008	0.045 *	0.580
	Control	13.68 (1.99)	12.92 (1.86)	−0.674	0.500	0.275			
Temperature (°C)	NFB and BFB	32.51 (1.19)	33.30 (1.55)	−1.826	0.068	0.745	−1.470	0.142	0.424
	Control	30.67 (1.95)	30.24 (3.36)	−0.944	0.345	0.385			
Heart rate (beats/min)	NFB and BFB	80.42 (9.80)	72.55 (2.94)	−1.992	0.046 *	0.813	−1.095	0.273	0.316
	Control	76.34 (13.75)	76.77 (9.88)	−0.405	0.686	0.165			
SDNN (ms)	NFB and BFB	68.98 (17.31)	95.21 (23.77)	−2.201	0.028 *	0.900	−2.008	0.045 *	0.580
	Control	77.85 (18.92)	83.50 (7.20)	−0.944	0.345	0.385			
LF/HF	NFB and BFB	2.10 (1.33)	3.67 (4.54)	−0.314	0.753	0.128	−0.183	0.855	0.053
	Control	1.47 (0.77)	1.88 (1.42)	−0.674	0.500	0.275			
pNN 50 (%)	NFB and BFB	10.59 (3.70)	12.83 (5.54)	−1.363	0.173	0.556	−1.095	0.273	0.316
	Control	13.02 (1.57)	12.83 (2.96)	−0.135	0.893	0.005			
Theta/SMR (uV)	NFB and BFB	2.78 (0.62)	2.29 (0.58)	−2.201	0.028 *	0.900	−2.008	0.045 *	0.580
	Control	2.78 (0.39)	2.67 (0.41)	−0.135	0.893	0.005			
Number of correct operations	NFB and BFB	32.67 (7.97)	48.67 (12.86)	−2.207	0.027 *	0.901	−2.562	0.010 *	0.740
	Control	30.50 (7.01)	34 (6.74)	−1.095	0.273	0.447			
% of incorrect operations	NFB and BFB	10.88 (6.86)	3.33 (3.08)	−2.023	0.043 *	0.826	−2.287	0.022 *	0.660
	Control	9.14 (8.46)	9.44 (6.90)	−0.365	0.715	0.149			

SD: Standard Deviation; * *p*-value < 0.05.

**Table 6:** Pre- and post-intervention effects of the dual-task performance.

Variables				Within Group Comparison			Between Group Comparison		
		Pre Mean (SD)	Post Mean (SD)	Z	*p*-Value	Effect Size	Z	*p*-Value	Effect Size
Number of taps	NFB and BFB	36.67 (4.68)	38.17 (3.43)	−1.063	0.288	0.434	−0.275	0.783	0.079
	Control	30.67 (6.65)	28.800 (11.69)	−0.730	0.465	0.298			
Reaction time (ms)	NFB and BFB	668.46 (97.98)	521.87 (54.79)	−2.201	0.028 *	0.899	−0.548	0.584	0.158
	Control	873.53 (290.46)	1039.53 (817.73)	−0.730	0.465	0.298			
Ratio tap/reaction time (ms)	NFB and BFB	0.06 (0.02)	0.07 (0.01)	−2.201	0.028 *	0.899	−0.730	0.465	0.211
	Control	0.04 (0.02)	0.04 (0.03)	0	1	0			

SD: Standard Deviation; * *p*-value < 0.05.

**Table 7:** Results of cognitive anxiety, somatic anxiety, self-confidence, and anxiety state variables pre and post intervention.

Variables				Within-Group Comparison			Between-Group Comparison		
		Pre Mean (SD)	Post Mean (SD)	Z	*p*-Value	Effect Size	Z	*p*-Value	Effect Size
Cognitive anxiety	NFB and BFB	2.13 (0.10)	1.60 (0.25)	−2.214	0.027 *	0.904	−0.746	0.456	0.215
	Control	2.33 (056)	1.84 (0.65)	−1.219	0.223	0.498			
Somatic anxiety	NFB and BFB	1.93 (0.33)	1.52 (0.53)	−1.997	0.046 *	0.815	−2.216	0.027 *	0.640
	Control	1.57 (0.43)	1.86 (0.66)	−1.342	0.180	0.548			
Self-confidence	NFB and BFB	3.23 (0.41)	3.83 (0.32)	−2.032	0.042 *	0.830	−1.878	0.060	0.542
	Control	3.27 (0.59)	3.54 (0.46)	−0.447	0.665	0.182			
State anxiety	NFB and BFB	22.17 (3.97)	10.83 (8.38)	−2.201	0.028 *	0.899	−1.742	0.081	0.503
	Control	16.17 (5.78)	16 (11.98)	−0.406	0.684	0.166			

SD: Standard Deviation; * *p*-value < 0.05.

**Table 8:** Results of flights on temporal, frequency, and non-linear measures of HRV.

Variables				Within-Group Comparison			Between-Group Comparison		
		Pre Mean (SD)	Post Mean (SD)	Z	*p*-Value	Effect Size	Z	*p*-Value	Effect Size
Mean HR	NFB and BFB	120.46 (20.42)	111.80 (15.35)	−0.365	0.715	0.182	−0.577	0.686	0.204
	Control	101.93 (14.15)	115.08 (12.09)	−1.826	0.068	0.913			
Mean RR	NFB and BFB	526.30 (89.01)	563.54 (77.70)	−0.730	0.435	0.365	−1.155	0.248	0.408
	Control	616.68 (91.48)	533.39 (57.28)	−1.826	0.068	0.913			
SDNN	NFB and BFB	40.11 (16.21)	70.61 (47.93)	−1.461	0.144	0.730	−2.021	0.043 *	0.714
	Control	73.75 (39.78)	43.51 (14.01)	−1.826	0.068	0.913			
RMSSD	NFB and BFB	14.10 (7.32)	28.21 (20.40)	−1.461	0.144	0.730	−1.443	0.149	0.510
	Control	33.91 (21.68)	16.22 (8.87)	−1.826	0.068	0.913			
pNN50	NFB and BFB	2.14 (2.25)	7.99 (7.87)	−1.461	0.144	0.730	−1.732	0.083	0.612
	Control	11.46 (9.77)	3.44 (4.97)	−1.826	0.068	0.913			
pVLF	NFB and BFB	5.53 (0.93)	6.44 (1.56)	−0.365	0.715	0.182	−0.189	0.773	0.067
	Control	4.90 (1.44)	5.02 (2.07)	−0.365	0.715	0.182			
pLF	NFB and BFB	74.29 (2.39)	69.95 (6.92)	−0.730	0.435	0.365	−1.443	0.149	0.404
	Control	71.69 (7.86)	73.95 (5.77)	−1.461	0.144	0.730			
pHF	NFB and BFB	20.19 (2.30)	23.60 (7.24)	−0.730	0.465	0.365	−1.443	0.149	0.404
	Control	23.40 (6.99)	21.02 (6.69)	−1.461	0.144	0.730			
LF/HF	NFB and BFB	4.53 (0.81)	4.12 (1.21)	<0.001	1	<0.001	−0.866	0.386	0.306
	Control	4.01 (0.98)	4.57 (1.41)	−1.826	0.068	0.913			
SD1	NFB and BFB	9.97 (5.18)	19.95 (14.43)	−1.461	0.144	0.730	−1.443	0.149	0.404
	Control	23.40 (6.99)	11.47 (6.27)	−1.826	0.068	0.913			
SD2	NFB and BFB	55.79 (22.33)	97.60 (66.14)	−1.461	0.144	0.730	−2.021	0.043 *	0.714
	Control	101.33 (54.17)	60.29 (18.95)	−1.826	0.068	0.913			
SampEn	NFB and BFB	0.80 (0.12)	0.86 (0.24)	−0.730	0.465	0.365	−0.577	0.564	0.204
	Control	1.01 (0.17)	0.79 (0.13)	−1.604	0.109	0.802			
DFA	NFB and BFB	1.12 (0.07)	1.09 (0.10)	−0.730	0.465	0.365	−1.155	0.248	0.408
	Control	1.01 (0.08)	1.08 (0.09)	−1.069	0.285	0.534			

SD: Standard Deviation; * *p*-value < 0.05.

## Data Availability

Data available on request due to restrictions (privacy of the pilots).
